# Application of a Multimedia Service and Resource Management Architecture for Fault Diagnosis

**DOI:** 10.3390/s18010068

**Published:** 2017-12-28

**Authors:** Alfonso Castro, Andrés A. Sedano, Fco. Javier García, Eduardo Villoslada, Víctor A. Villagrá

**Affiliations:** 1 Dpto. Ingeniería de Sistemas Telemáticos, Universidad Politécnica de Madrid, 28040 Madrid, Spain; villagra@dit.upm.es; 2 Telefónica Investigación Desarrollo, 47151 Valladolid, Spain; andresalberto.sedanofrade@telefonica.com (A.A.S.); eduardo.villoslada@telefonica.com (E.V.); 3 Telefónica Investigación y Desarrollo, 28050 Madrid, Spain; fco.javier.garciaalgarra@telefonica.com

**Keywords:** Autonomic Communication, self-provisioning, knowledge-based management

## Abstract

Nowadays, the complexity of global video products has substantially increased. They are composed of several associated services whose functionalities need to adapt across heterogeneous networks with different technologies and administrative domains. Each of these domains has different operational procedures; therefore, the comprehensive management of multi-domain services presents serious challenges. This paper discusses an approach to service management linking fault diagnosis system and Business Processes for Telefónica’s global video service. The main contribution of this paper is the proposal of an extended service management architecture based on Multi Agent Systems able to integrate the fault diagnosis with other different service management functionalities. This architecture includes a distributed set of agents able to coordinate their actions under the umbrella of a Shared Knowledge Plane, inferring and sharing their knowledge with semantic techniques and three types of automatic reasoning: heterogeneous, ontology-based and Bayesian reasoning. This proposal has been deployed and validated in a real scenario in the video service offered by Telefónica Latam.

## 1. Introduction

The growth of TV channels, series and movies that people watch as well as the greater and better coverage of sports reveal that we are living the golden age of TV. Since its birth, TV has evolved from a broadcast, family oriented product to another one on demand and online that can be individually consumed, i.e., now every consumer watches what he/she wants, when he/she wants and where he/she wants. Furthermore, new devices for video consumption, such as desktops, laptops, tablets or smartphones, have not killed TV. On the contrary, the TV set is still the main screen where people watch video content.

Telecom Operators include video services in their commercial offer, such as Verizon with FIOS, AT&T with DirectTV or Telefónica with Fusión TV. 

Global Video Platform (GVP) is Telefónica’s global multi-technology and multitenant video platform powering TV services across the company footprint. As of July 2016, GVP is deployed across different Operational Business units (OB) in Spain, Brazil, Argentina, Chile, Perú, Nicaragua, Costa Rica, Guatemala, El Salvador, Uruguay and Colombia.

Video services are complex sets of technological and organizational parts. Each one is managed by different teams with different roles such as content or infrastructure provider, device manufacturer and so on. These organizations are associated with administrative domains included in the different OBs. Video services are powered by multiple technologies (IPTV, OTT, CDN, etc.) with several streaming formats, such as smooth streaming or HLS, both for Video on Demand (VoD) and Live Broadcasts. Thus, these inter-domain video service environments have a very high complexity which leads to a real challenge for a proper service management and operation.

Regarding the current state of the art on this area, nowadays there are many commercial products that can provide different tasks related to each aspect of the service and operation management (incident management, fault diagnosis, SLA management, etc.), but in an isolated way. There is a lack of proposals that can be directly applied to the problem: global (instead of isolated functionalities) and integrated service management for a complex service, in an intelligent and cooperative way over an inter-domain service deployment. This approach would link the business vision with the operational vision, covering management aspects such as SLA, fault diagnosis or incident management.

This paper proposes an extended architecture that provides a global solution of service management, linking and integrating different service management functionalities, such as SLA, fault diagnosis or incident management. It includes a specific proposal for the integration of fault diagnosis with the other service management functionalities in a global video service environment. 

To solve the fault diagnosis problem, this extended architecture includes a specific Multi-Agent System with Bayesian reasoning [1], which has been applied to video service management. The integration with the other service management functionalities is provided with an ontology-based global reasoning agent system. This paper extends and validates the architecture for global management services introduced during previous works [2].

The proposed architecture has been deployed and validated in a real Telefónica’s online video service. The evaluation has been done with data collected during the last eighteen months of operation. The results show a significant improvement in service management, and a reduction in the incident solution time.

The paper structure is the following: Section 2 discuss other proposals and initiatives related to the problems presented in this paper. Section 3 provides an overview of the proposed agent’s architecture for the video service management scenario. Section 4 describes the real scenario where the system has been deployed. Section 5 outlines the results of the system validation in that real scenario. Finally, Section 6 provides conclusions and discusses potential future work.

## 2. Related Work 

Both research community and industry has devoted considerable effort to overcome the complexities in the service management area. Some of these industry efforts have been oriented to the standardization field, pointing out as the most popular standards ITIL [3] and eTOM [4]. The Information Technology Infrastructure Library (ITIL) is a set of concepts and policies for managing IT infrastructure, development and operations that has wide acceptance in the industry. The eTOM (enhanced Telecommunication Operations Map) is the framework proposed by TMF (Telecommunication Management Forum) which describes all the enterprise processes required by a service provider, analyzing them in different levels of detail according to their significance and priority for the business. 

On the other hand, research community has provided many valuable contributions to different technological areas of network and service management. Network management based on ontologies are a representative example. De Vergara, J.E.L. et al. [5] present an in-depth study on the application of semantic technologies to the network management domain. Lasierra, N. et al. [6] propose a combination of the autonomic computing paradigm with ontologies to be able to solve the integration of management data. Keeney, J. et al. [7] present a solution for the management of complex IT services composed by different sources, which are heterogeneous and geographically distributed. 

Other works have combined standardization and ontologies techniques for solving the complexity of the service management. Valiente et al. [8] design an ontology representing the best practices of the management processes based on ITIL V3. Wong, A. et al. [9] provide a similar work taking eTOM as service process framework. Stamatelatos et al. [10] present an information model for the management of the future internet based on TMF Information Framework (SID).

The complexity of video services has increased in recent decades. However, methods of managing these services and handling their faults have not kept pace. Although there are good commercial solutions for service integral management, such as IBM Tivoli [11], HP Network Management Center [12] or Spectrum Service Availability [13], their deployment requires a high effort of integration with the service platforms and networks. However, the global management system proposed in this paper allows a simple integration of all operational tasks included in the service management area.

In the area of fault diagnosis, most of the operations are still based on manual processes. Among the current proposals to help in this task, the use of Multi Agent Systems (MAS) for network diagnosis, which has been previously proposed by several researchers, is remarkable [14,15,16]. Other proposals (such as by Martin et al. [17]) present a framework based on intelligent agents for network management that uses rule-based reasoning. Telefónica has largely used rule-based systems, but rule-based maintenance has been an issue due to the high number of manual changes needed in the rules in a daily operation. The use of Bayesian networks has considerably reduced the required effort, and introduces capabilities of learning and uncertainty management. Both Leitao et al. [18] and Mendoza et al. [19] present a MAS focused on the configurability of the system for collaborative tasks using adaptable agents. In the evaluation of the architecture presented in this paper, we have defined agents with specialized roles, although in future research we will evaluate whether the service management functionality can be improved by allowing all the agents to be adaptable and able to play different roles. Luo et al. [20] proposed a fault diagnosis system using Dempster–Shafer evidence theory [21,22] with combination rules to solve the possible information conflicts from multi-sensors systems. 

In a previous work [1], we proposed an architecture with a Multi Agent System based on an extended Belief–Desire–Intention (BDI) model that combines heterogeneous reasoning processes, ontology-based reasoning and Bayesian reasoning. The architecture was deployed at Telefónica O2 Czech Republic to manage an Internet Business service but only in a controlled environment and with a reduced number of service management areas. The present work extends this architecture so it can deal with a more realistic and complex functionality: a global video service provisioning. The architecture has been successfully deployed and validated in a real production environment. 

In addition, this paper leverages previous contributions [2], where we defined a model based on ontologies to manage the service. The approach presented in this paper goes a step beyond from the previous work, designing and validating a whole service management model in a real scenario: the management of a global video service of Telefónica. This work integrates different aspects of service management as fault diagnose with quality, problem and SLA management. The previous model has been extended also by applying Bayesian networks to handle the uncertainty of the incomplete or unreliable data. 

## 3. Architecture Framework

This section describes the proposed agent architecture for a whole service management. The approach used is aligned with the Multi agent architecture paradigm.

### 3.1. Problem Description

A multi-agent architecture is made up of a set of heterogeneous agents working in a coordinated way to obtain a common goal. The main agent’s characteristics are its autonomy, social ability, reactivity, proactivity, mobility, temporal continuity, adaptability and learning [23,24,25]. 

The success of such architectures is their ability to synchronize the performance of tasks assigned to each agent. For that, agents exchange their knowledge and beliefs with the rest [26,27,28,29,30].

Therefore, the inherent complexity of Multi-Agent Systems should be adapted and customized to solve the inter-domain complexity needed for a global video service management, as introduced in Section 1.

There are two main challenges that need to be addressed by a multi-agent based architecture such as the one presented in this paper:

- Uncertainty in the diagnostic process: At the beginning of the diagnostic process, only partial information is known, and it is not feasible to collect or even model all the available information due to the complexity of the environments. Thus, diagnosing complex services requires reasoning under uncertainty.
- Government of the whole service management: There will be a set of distributed agents with a collaborative way of working. Agents are distributed across different environments, both technological and administrative, and dedicated to different management services, such as fault diagnosis or problem management. Each agent is associated with one management function on a specific domain. In addition, each agent will have an associated knowledge plane of its domain and will have the capability to share its experiences of its environment with the rest of the agents.

### 3.2. General Architecture

**Figure 1:**
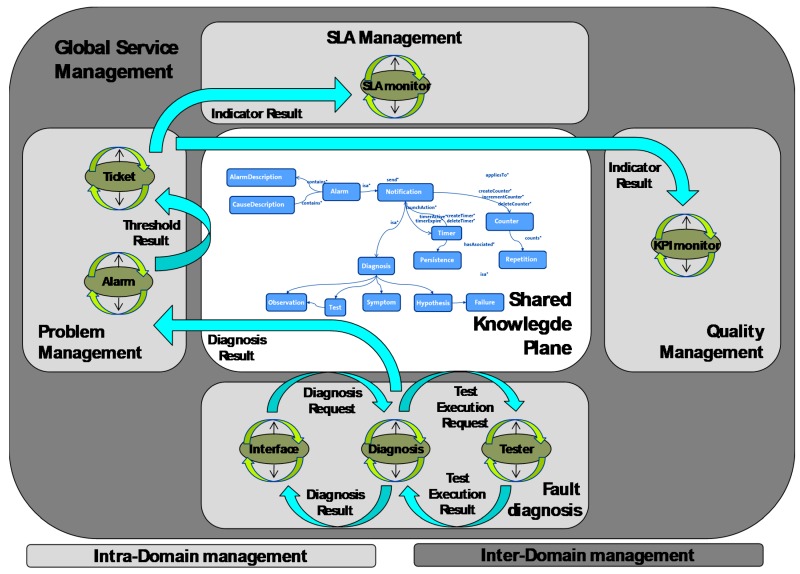
Agent system structure.

Figure 1 shows the Multi-Agent System architecture. It depicts the distribution of the agents for the different domains (technological or administrative) and operational tasks (fault diagnosis, service problem management, etc.). The management of the service is done at two levels: one associated with each operational task (intra-domain) and another that links all the operational tasks, providing a global service management (inter-domain).

An extended MAPE-K (Monitor, Analyze, Plan, Execute—Knowledge) model [31], based on previous work [32], has been designed to govern the architecture. It meets the requirements discussed above such as coherent distributed reasoning and managed shared knowledge. The role of the managed element for the MAPE-K model is assumed by the service for this extended model. Another assumption in the extended model is about the execution phase: usually, the MAPE-K loop acts by modifying the configuration of a managed element. Nevertheless, our extended model replaces the managed element for a managed service modifying its management properties (i.e., generating a new ticket).

The key element of this architecture is Knowledge. It includes the description of the service domain for each agent based on an ontology. This ontology includes a conceptual model with concepts, relations and individuals. This knowledge can be modified when the environment conditions change due to external causes or new knowledge inferred by the agents during the execution phase. 

Each agent has an associated knowledge plane that includes all the conditions for the service regarding its domain. The set of knowledge planes is called Shared Knowledge Plane (SKP) which is the fundamental element of the above model. It includes the common knowledge for all the domains and governs the agents’ behavior. It also stores the semantic model, the set of instances and the reasoning rules. The fact that the domain description and management rules are enclosed within the ontology reduces the complexity of the agents. The SKP provides the way of exchanging knowledge between agents. Technically, the agents read or update information ontologies that are published in a web server with a mechanism similar to web 2.0 Wikis. This functionality is based on the work performed in [33].

### 3.3. Reasoning Techniques

To find the more suitable reasoning techniques to be applied to these scenarios, where an interworking of inter-domain and intra-domain service management is needed (as shown in Figure 1), Table 1 presents a comparison of some of them in accordance with Zhang [34]. Comparing alternatives such as reasoning offered by Bayesian Networks (BNs), rule-based or case-based reasoning, Bayesian inference performs better on incomplete datasets and in the presence of uncertainty while maintaining coherence and consistency. Moreover, Rule-based reasoning enables a more expressive method of inference when reasoning in event-based systems with cause-effect relations clearly defined.

The reasoning technique chosen to meet the first challenge is the probabilistic reasoning offered by Bayesian Networks (BNs) in a distributed way (Multiply Sectioned Bayesian Networks (MSBNs) [35]). Thus, agents that handle uncertainty use a Causal Model (implemented as a BN). This BN enables distributed causal inference to update beliefs. The Causal Model is a subnet that is part of the MSBN, allowing to keep coherence during the distributed reasoning process.

Rule-based reasoning technique has been chosen to meet the second requirement about the government of the whole service management. Using this approach, each agent contains a rule engine and, therefore, its behavior is reduced to perform rule based inference. 

As was already described in Figure 1, the proposed architecture for the video service is composed of two main modules:

- The inter-domain management module provides global video service management and is based on Ontology-based Reasoning Module (see Section 3.1.).
- The intra-domain management module is based on Distributed Bayesian reasoning (see Section 3.2.). Fault diagnosis is an intra-domain management module for this paper.

### 3.4. Inter-Domain Management. Ontology Modelling and Reasoning

A Shared Knowledge Plane [32] provides government of the whole service management. It contains the semantic model, the set of instances and the reasoning rules offered to the agents to rule their behavior. The fact that domain description and management rules are enclosed within the ontology reduces the complexity of the agents. 

**Figure 2:**
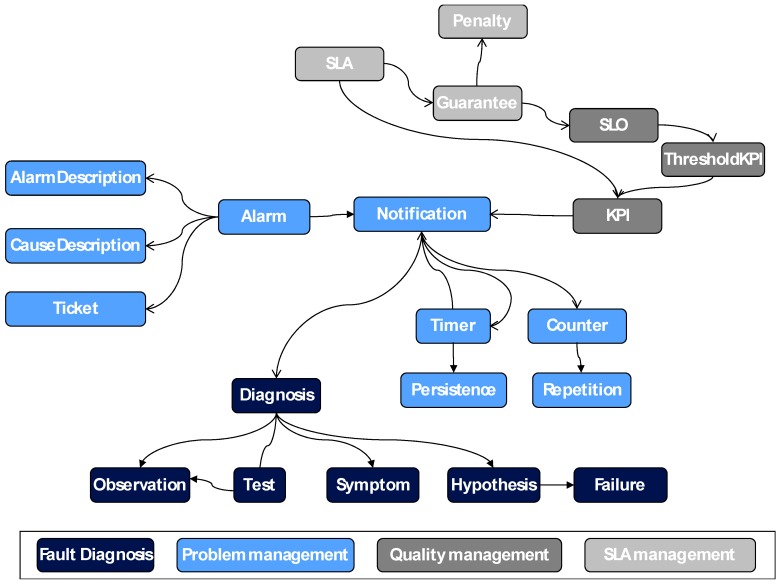
Whole management ontology.

The concepts of this ontology are based on several management aspects which comprises the set of functions aiming to detect, isolate, correct and report a malfunctioning in video service. The main functions are fault diagnosis and problem, quality and SLA management. Figure 2 presents these concepts and relationships in this global shared ontology regarding whole management, with different colors depending on their service management functionality.

A set of rules has been defined to govern the whole service management. When a malfunction is detected, these rules trigger the implementation of preventive and corrective actions related to the generation alarm into problem management, tracking Key Performance Indicators (KPIs) or meeting of SLA including the penalties management.

### 3.5. Intra-Domain Management Apply to Fault Diagnosis

We extended the standard MAPE-K control loop [31] to provide coherence and consistency in both causal distributed reasoning and rule-based reasoning. Agents execute the control loop and interact with the reasoning modules. 

As described previously, Bayesian reasoning copes with uncertainty in the process of diagnosis. The Bayesian Network design follows the BN3M [35] model, which classifies network nodes (variables) into three groups: evidence, root causes and context (including auxiliary variables). Once the variables of one domain are identified, the next step is defining their connections through a Conditional Probability Table (CPT).

These probabilistic relationships (domain knowledge) may be elicited from human experts, data-mined from historical data or with a mixed process.

During the diagnosis procedure, the causal model is used in the hypothesis generation step. Available information is added as soon as possible to the BN (symptoms, tests results, context variables, etc.). These data are fed as evidence.

Each time a new piece of evidence becomes available, the Bayesian Reasoning Module yields a set of hypotheses with their degrees of confidence. The MSBN technique allows for several agents to reason concurrently with their own local Bayesian Network and then share their findings. This requires causal models to be properly connected. Some variables of the Bayesian Networks behave as bridges for connecting the local Bayesian Network, and an initialization and update algorithm is used to maintain coherence among the subnets, as detailed by Xiang et al. [35].

The complete government of the fault diagnosis intra-domain can be included in the different concepts of the previous ontology (Figure 2): each diagnosis has a body of evidence (symptoms and test results, referred to as observations) and hypotheses of failure. These hypotheses will be proposed by performing a set of tests about the environment and obtaining information that could generate different hypothesis. Depending on the available information, the agent will select the next test to perform to get a quick and reliable diagnosis.

The acquisition of the knowledge from the causal model is performed to create individuals of the ontology following these steps:

1. Each time a diagnostic procedure starts, individuals for this ontology are extracted from the causal model (Knowledge Base).
2. All nodes of the causal model can be classified into one of three types mentioned above for the BN3M model: evidence, fault root causes and context. Initially, individuals representing symptoms are created based on the information received in the diagnosis request message and individuals representing hypotheses are extracted from the causal model based on the nodes that represent fault root causes.
3. All individuals representing tests are extracted to analyze which evidence can be used in the causal model.
4. At this point, the hypothesis confirmation loop begins. Each time a new test result is available, a new observation individual appears to represent the result, and the test is marked as performed. Bayesian inference runs again and a new set of updated hypotheses replace the previous set.
5. When all possible tests are run or one or several hypotheses reach a threshold, the diagnostic process is complete and the final hypotheses are returned to the agent that requested the diagnosis.

## 4. Case Study

This section describes the practical experience in the application of the service management Multi Agent System proposed in the previous sections. This system has been deployed at Telefónica to manage the global video service. A Multi Agent System, based on the described architecture, has been installed to diagnose service failures. In addition, a set of agents are providing different aspects of the whole service management as sources for the diagnosis information. The video service environment is described in Section 4.1, the deployed agents and the specific features developed to adapt the model to the client requirements are presented in Section 4.2.

### 4.1. Video Service Environment

The environment where this agent system has been deployed is Telefónica’s global video service. As this service has many features, automatic diagnosis is available right now for the Over the Top (OTT) service with both Video on Demand (VoD) and Live broadcast (LiveTV) streaming technologies.

The fault diagnosis management is deployed in the live environment while the whole video service management is deployed in pre-production environment. Both environments, live and pre-production, have interfaces to link the whole video service management with the fault diagnosis.

The video service architecture is a blend of global and local components. Global components support functionalities for all the OB (Operational Business unit) while local ones only support functionalities for one OB. There are two different types of global components: those with a regional distribution, such as CDN (Contact Delivery Network), and global with local distribution, such as connector with the BSS (Business System Support) of the OBs. 

**Figure 3:**
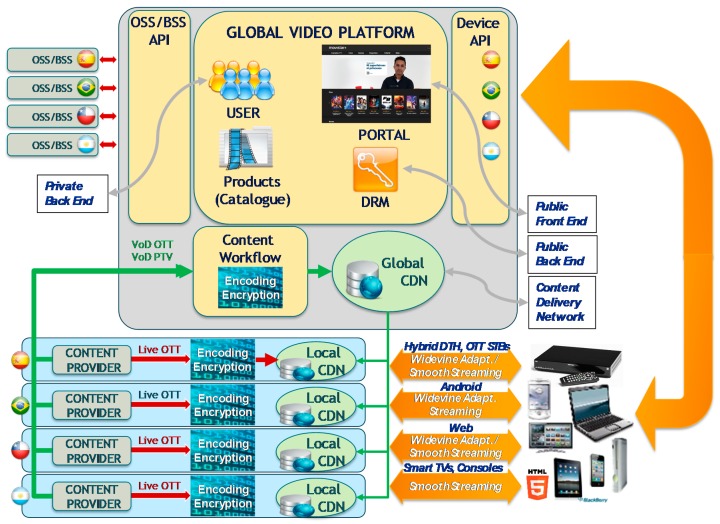
Video service environment.

A simplified diagram of the video service architecture contains the following elements, as show in Figure 3:

- Public Front End: Web Portals, public Interfaces, etc. for devices.
- Public Back End, logic accessed from devices: user login and trusted devices.
- Private Back End, logic accessed from OSS/BSS system: register users, etc.
- Content Delivery Network (CDN), to distribute the media content such as videos and covers using distributed DNS service.

For VoD OTT, local content providers ingest the content to the global elements where the signal is encoded and encrypted. Then, the signal is sent to the global CDN element (Origin) and distributed to the local CDN elements (proxy origin and end points). 

The existence of different versions of customer applications (Android, IOS) requires publishing different API releases, adding complexity to the service.

When a device wants to begin a video session, it connects to the suitable portal allocated in the global video platform (Public Front End) and security information is exchanged with the Public Back End. Then, the device requests both the VoD and LiveTV signal, and a connection with the local CDN element is set up. 

An important issue is the latency with Front End and Back End from each region: a distributed diagnosis system assists in monitoring this latency. Data required to carry out a diagnosis in this scenario are geographically distributed. Moreover, information may be missing, unreachable (due to connectivity problems) or even outdated. Thus, the application of uncertainty reasoning techniques is almost mandatory for fault diagnosis management. 

As a key management functionality in the whole video service management, diagnosis fault automation will improve the values of a set of video operational indicators. Thus, Mean Time to Diagnose (MTTD) will decrease, the ratio proactive/reactive incidents will improve, anticipating to customer complaints and reducing the incident resolution time.

### 4.2. Service Management MAS Development and Deployment

Following the model presented in Section 3, a Multi Agent System for video service management has been deployed in different points of the video service infrastructure. The system was developed using the JADE platform [36], which offers an open agent model compatible with the proposed extended MAPE-K model. 

#### 4.2.1. Fault Diagnosis Environment

For the failure diagnosis management, the interaction flow starts with the Benjamins model of diagnosis [37]. There are three steps in any diagnosis scenario: (a) symptom detection; (b) hypothesis generation; and (c) hypothesis confirmation. Each time a new symptom is present, it triggers a set of hypotheses. This set contains possible root causes. Then, each hypothesis must be confirmed or discarded to reach reliable conclusions. A list of hypotheses ordered from highest to lowest probability is generated, discarding the lowest ones. Tests are executed to determine the state of the system and the results are fed back to the hypothesis generation step to update the current set of hypotheses with the available knowledge. 

**Figure 4:**
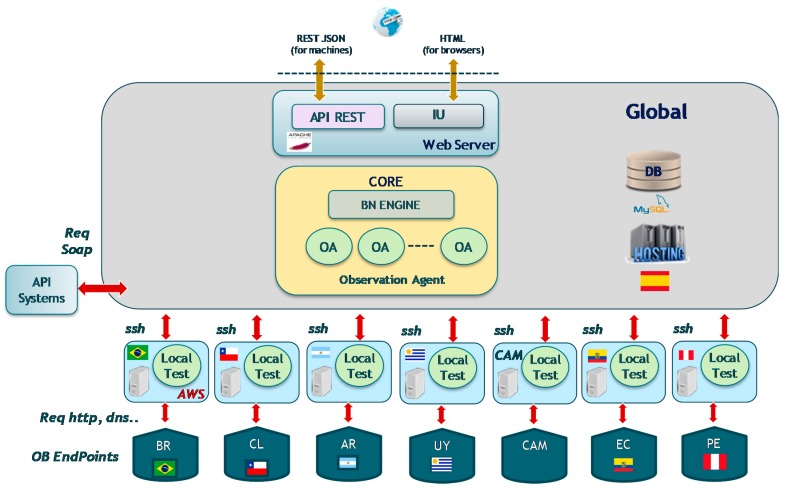
Diagnosis architecture.

Figure 4 shows the geographically distribution of the Diagnosis Agents. In video service production environment, we identified the different several diagnosis scenarios depending on the local infrastructures. There is one Diagnosis Agent for each diagnosis scenario. Each Diagnosis Agent includes its own Bayesian Network (BN).

As shown in Figure 4, the Core subsystem of the global site orchestrates the diagnosis tests in the local sites. The local sites for the failure diagnosis system are connected with Local CDN nodes (End Points), launching http sessions and using local DNS services. In addition, the global site can connect to other global service management systems (OSS/BSS) via SOAP. The global site provides additionally two web service interfaces: one for programmatic access based on API REST and the other one for the maintenance users based on HTML.

The system is dimensioned to support all the observation requests to get a complete diagnosis without performance problems. Both sites can be implemented in physical or virtual machine. 

The minimum requirements for the HW, SW and connectivity for global and local sites are included in Table 2.

There are three different types of agents: interface, diagnosis and Test Agents.

Interface agents launch the fault diagnosis. There are two ways to launch a service diagnosis: manually, by human operators, or, more often, automatically. Thus, every m minutes, an automatic diagnosis checks the end to end service scenario status.

Each observation is assigned to a Test Agent. The tests require a set of parameters dynamically computed, given the feature of the video service. An example of this is content switch. Before checking typical user actions, there is a query to the movie catalog that identifies the proper movies to use in diagnosis tests. 

Once the required information is available, the Diagnosis Agent collects the observations from the Test Agents. There are four kinds of Test Agents:

- Portal test connects to the endpoint and checks that it is up and running; this type of test is used with several endpoints, depending on the OB, the kind of device and its version.
- Public Back End test executes requests flow for each main user action (Home loading, Movie and Series detail, and Purchase Movie)
- CDN test: As the proper use of CDN is based on change in DNS resolutions (with reduced Time to Live parameters), the test over the CDN content has to adapt to it. Another relevant feature to consider in CDN tests is the proximity with end users: as CDN responses depend on geographic location, the monitoring is locally performed in each region.
- Private Back End test: In this case, the tests are based on administrative tasks. These tests check the logic accessed to OSS/BSS systems as registered users.

When a test is performed, the result is fed back to the causal model and Diagnosis Agent. Each test has a timeout to execute or it will be marked as Failed (Unknown).

**Figure 5:**
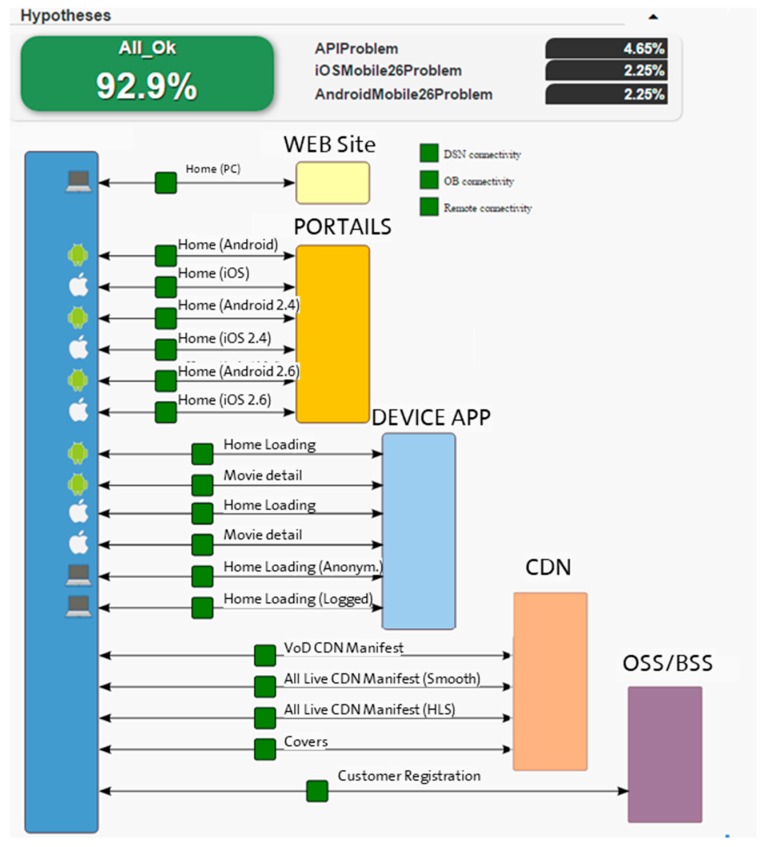
Diagnosis results graph.

Diagnosis results are displayed for the human operator. Figure 5 shows the results of the observations, which are based on the diagnosis.

This diagnosis result graph is easy to see, with a color code: OK tests (green), NOK (red), neither OK nor NOK (orange) and Unknown tests (grey). In the example shown in Figure 5, all results of the observations are OK. Hypotheses are retrieved with a percentage that marks the diagnosis certainty. In this case, the retrieved hypothesis is “ALL_OK” with a probability of 92.9%.

The Bayesian Network defines complex relations between hypothesis and observation nodes. These relations are based on links between the nodes and the assignation of a set of weighted values for each link. The number of observations ranges from 25 to 60. This Bayesian Network is supervised and the weighted values of the links are constant while the scenario is not modified. Thus, there are no important performance requirements associated with the BN.

#### 4.2.2. Whole Service Management Environment

The whole service management is governed by the Share Knowledge Plane, which orchestrates the activities of different management aspects based on a semantic model.

The ontology has been written in OWL language. Protégé 3.4 [38] has been used to generate the ontology and behavior rules are written in SWRL (Semantic Web Rule Language). Each agent uses the Java library Jena 2.5 [39] to perform the parsing and manipulation of the ontology information together with the Pellet reasoning engine to execute the rules.

**Figure 6:**
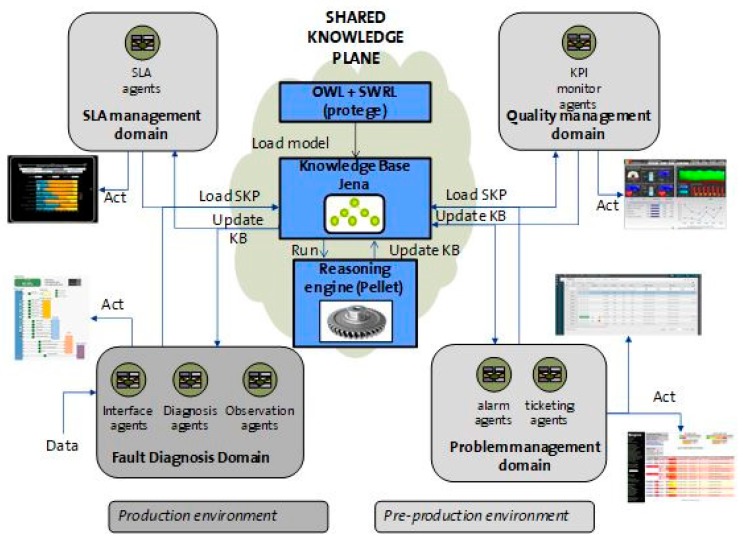
Shared Knowledge Plane schema.

The result of each observation is sent to the SKP and stored in the knowledge base of the system (Figure 6). The remaining agents are notified of the change in the knowledge base and each one executes its own routine. Hence, an incident could be opened in the trouble ticketing tool or an alarm could be dispatched to the supervision system. The incident characteristics, such as severity and the assigned resolution team, are part of the ticket information. Quality management agents are continuously extracting data to assign values to a set of indicators shown in operational dashboards. SLA management agents collect information to alert of breaches of the agreements.

The detailed steps for the complete use cases are listed below:

- Define the semantic model (OWL + SWRL) of the Shared Knowledge Plane and generation of the OWL text file using protégé.
- Create the Knowledge Base (KB) in the Shared Knowledge Plane based on the OWL + SWRL model.
- Update the KB in the Shared Knowledge Plane with the received information from any domain.
- If necessary, execute an inference cycle reasoning in the SKP, using the Pellet inference engine.

## 5. Results

The system was evaluated during the first half of 2016. During this time, the service management MAS was operating and recording data on diagnosis cases. The system performed around 100,000 automatic diagnoses, from three countries (Argentina, Brazil and Spain). At the same time, this information was sent to the preproduction environment to be used by the agents that integrate the whole video service management. 

The evaluation methodology consisted of two steps. First, the coverage of the dataset relative to the global problem was analyzed to check whether these data are sufficiently representative as explained below. Then, several KPIs were defined to evaluate the business benefits of the system.

### 5.1. Fault Diagnosis Metrics

**Figure 7:**
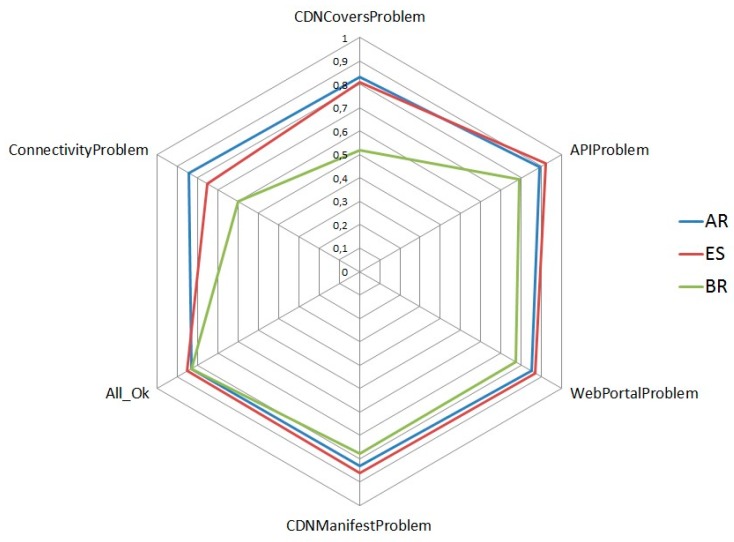
Normalized entropy of various root causes of faults.

To analyze the complexity of the scenario, the entropy of possible diagnosis cases has been calculated and compared with the entropy of each possible root cause. This entropy represents how the same fault root cause can be inferred in the environment for different test results. Many different test result sets can result from the same fault type. In this case, six different fault types have been identified for three different countries. The Bayesian network has been successfully used to reason under this uncertainty. The normalized entropy of each possible root cause was compared to determine which fault root cause (fault type) is more complex. Figure 7 shows that most fault root causes exhibit high entropy because these fault types can be shown as different symptoms and test results. In addition, different entropy results are showed by OBs due to different elements that compose the service in each OB.

**Figure 8:**
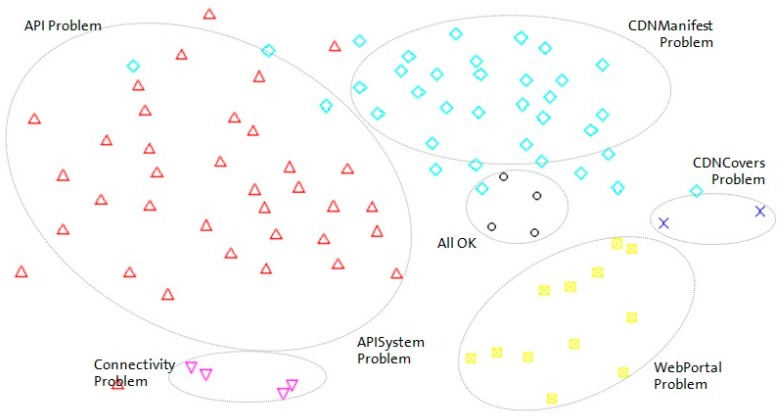
Fault root cause clusters.

To graphically represent all diagnoses stored in the database, a Sammon mapping algorithm [40] has been used to represent the diagnoses in a two-dimensional graph (see Figure 7). Using this algorithm, the relative Euclidean distance among all stored diagnoses is maintained. As shown in Figure 7, diagnoses with the same highest percentage hypothesis (i.e., the same most probable cause of failure) are close to one another in the graph (Figure 8). To highlight these clusters, each one has been rounded and labeled properly. Furthermore, to confirm that the Sammon mapping algorithm is a good way to represent the complexity of each fault root cause, the area of each region is directly related with its entropy (high entropy is represented with a large region). To interpret this graph, note that two diagnosis cases that are graphically in the same place in Figure 8 represent cases with the same symptoms and the same final hypotheses, i.e., the Euclidean distance between these cases is zero.

**Figure 9:**
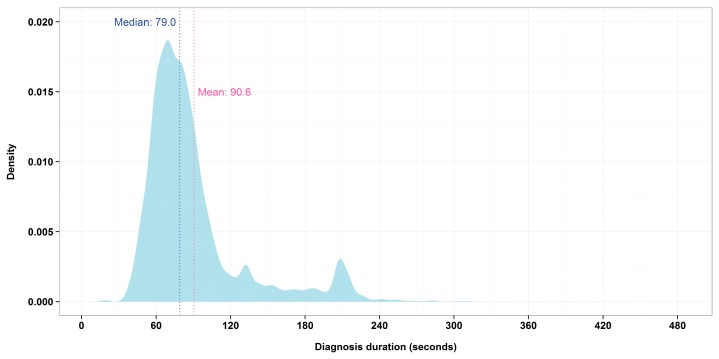
Density plot of diagnosis duration (in seconds).

Diagnosis time is presented in a density plot in Figure 9. Most of them have a value between 40 and 110 s. The system has two timeouts to limit the duration of the wrong observations so the distribution has two minor peaks around those values.

The histogram can be understood as a bimodal distribution with a mean value of 90.6 s and a median of 79.0 s.

### 5.2. Business KPIs

Several indicators were defined to assess the business benefits of the system. Some of these indicators are associated with the performance (KPIs) of the operational activities. The values of these KPIs improved after the deployment of this solution in video production environment. Others indicators are related to the service quality (KQI), and showed the evolution of some aspect of the customer experience. These indicators were measured between March and June 2016. 

KPI1 is the percentage of incidents opened in trouble ticketing by proactive way versus all the incidents after the system had determined a service malfunction. It is used to measure the usage of the system by human operators, i.e., the acceptance rate of the diagnosis system. It shows the user confidence in the system but when the whole service management was developed in production environment the tickets were automatically opened. It has been measured considering the values in the trouble ticketing tools in production. This KPI was initially 27.27% in March 2016, and, after the new set of hypotheses in the diagnosis system were included, it increased to 52.94% in June.

KPI2 is used to measure the percentage of incident solution time (i.e., the diagnosis and repair time) of the proactive incidents versus all the incidents. This indicator provides information about the improvement in the restoration time with a stable video platform version and a continuous improved hypothesis for the fault diagnosis. Initially, this KPI was 85.93% in March 2016 and it decreased to 5.29% in June 2016.

**Figure 10:**
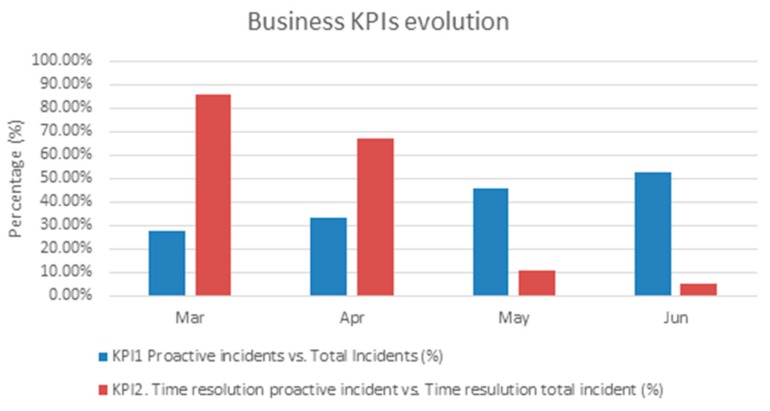
KPI monthly evolution.

Figure 10 shows the evolution of the both performance indicator during the evaluation period.

**Figure 11:**
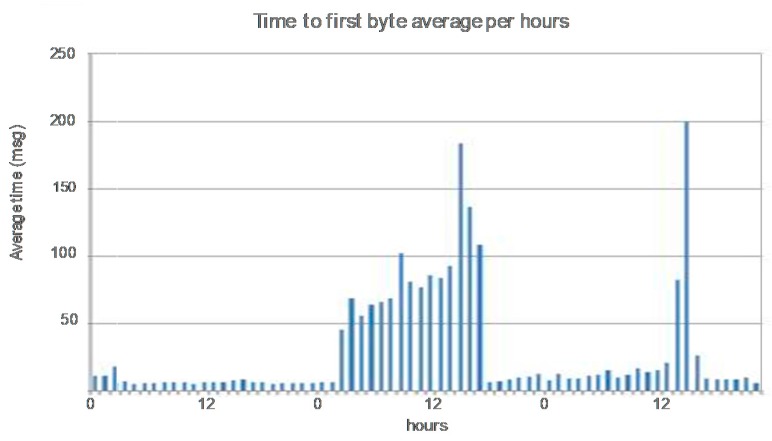
KQI evolution.

The use of the whole video service management in the pre-production environment has facilitated the procurement of indicators related to the quality of experience (QoE). “Short start-up time” is one of most important KQIs for the video on Demand service. Time to First byte is an observation collected by the system and provides information about the delay between the query of content and its reception. Thus, when the value is higher than a threshold, a warning about the quality perceived by the customer is triggered by the systems. Figure 11 shows the average evolution per hour of this indicator, in which the degradation of the short start up time for a determinate hour is shown. Early detection avoided the decrease of the quality of experience of the users. 

This evaluation validates the use of our solution for the fault diagnosis for the video service management. In addition, the successful result of the test in preproduction environment of the whole management system is the first step to its future incorporation to production environment. Thus, this system improves the operation, providing performance indicators and information about the quality perceived by the users.

## 6. Conclusions and Future Work

This article has proposed a Multi-Agent System (MAS) for fault diagnosis, which has been validated in a real scenario in the video service offered by Telefónica Latam. In addition, its integration with a whole video service management system based on this MAS and described in a previous work [2] has been validated in preproduction environment. The proposed system satisfies the main challenges identified in Section 3.1:

- Uncertainty in the diagnostic process: Including adequate reasoning techniques into a more effective diagnosis system will also increase customer satisfaction and decrease the human resources required for diagnosis tasks. In this sense, the MAS explained in this paper contributes to improve the effectiveness of the both fault diagnosis and whole service management.
- Government of the whole service management: The architecture expands from intra-domain to inter-domain management with a Shared Knowledge Plane that assures a common and consistent information model, which will allow a global service management as stated.

An important conclusion of this research work is that agent technology is suitable for distributed management, including fault diagnosis. Agent technology has proven to be very useful for adapting the identified roles to different domains without requiring extensive training.

The proposed MAS architecture is sufficiently flexible to enable progressive deployment of agents to cover all the management aspects. This strategy has also been a key in the success of the deployment of the system.

Now that the architecture has been validated in the scenario presented here, several possible paths can be explored. To get a higher degree of self-management, we are working on incorporating machine-learning techniques to forecast the behavior of the video service elements. This new explored area is based on the Shared Knowledge Plane, which provides a deep control of actions associated with the management aspects.

Another area of evolution of this architecture is the integration with different heterogeneous services, which can share the complexity of the global video service presented here, but rely on different fault diagnosis techniques and different KPIs. An adequate service candidate is the integration with the management of the so-called Internet of the Things, where the service management will extend to manage the different and heterogeneous devices included in client premises.

## Figures and Tables

**Table 1:** Reasoning techniques.

Reasoning Technique	Rules Systems	CBR	Fuzzy Logic	Bayesian Inference
Coherence/Consistency	Good	Good	Bad	Good
Handle uncertainty	Null	Null	Good	Good
Failures tolerance	Medium	Bad	Medium	Medium
Maintain private data	Good	Medium	Good	Medium
Incomplete dataset	Bad	Good	Medium	Good

**Table 2:** Resources requirement.

	Resource	Physical Machine	Virtual Machine
Global	HW	8 CPU cores	8 CPU cores
		16 GB RAM	16 GB RAM
		1 Tera HD	1 Tera HD
	SW	RedHat 6.4	
	Connectivity	300 Mbps	
Local	HW	4 CPU cores	4 CPU cores
		8 GB RAM	8 GB RAM
		120 GB HD	120 GB HD
	SW	CentOS 6.4/RedHat 6.4	
	Connectivity	10 Mbps/1 Mbps residential Internet access	
		(20 Mbps/2 Mbps or more recommended)	
		Static public IP or dynamic DNS service and NAPT/port forwarding for OAM access (SSH)	
